# Short Term Effects of Weather on Hand, Foot and Mouth Disease

**DOI:** 10.1371/journal.pone.0016796

**Published:** 2011-02-11

**Authors:** Yien Ling Hii, Joacim Rocklöv, Nawi Ng

**Affiliations:** 1 Umeå Centre for Global Health Research, Epidemiology and Global Health, Department of Public Health and Clinical Medicine, Umeå University, Umeå, Sweden; 2 Occupational and Environmental Medicine, Department of Public Health and Clinical Medicine, Umeå University, Umeå, Sweden; Duke-National University of Singapore Graduate Medical School, Singapore

## Abstract

**Background:**

Hand, foot, and mouth disease (HFMD) outbreaks leading to clinical and fatal complications have increased since late 1990s; especially in the Asia Pacific Region. Outbreaks of HFMD peaks in the warmer season of the year, but the underlying factors for this annual pattern and the reasons to the recent upsurge trend have not yet been established. This study analyzed the effect of short-term changes in weather on the incidence of HFMD in Singapore.

**Methods:**

The relative risks between weekly HFMD cases and temperature and rainfall were estimated for the period 2001–2008 using time series Poisson regression models allowing for over-dispersion. Smoothing was used to allow non-linear relationship between weather and weekly HFMD cases, and to adjust for seasonality and long-term time trend. Additionally, autocorrelation was controlled and weather was allowed to have a lagged effect on HFMD incidence up to 2 weeks.

**Results:**

Weekly temperature and rainfall showed statistically significant association with HFMD incidence at time lag of 1–2 weeks. Every 1°C increases in maximum temperature above 32°C elevated the risk of HFMD incidence by 36% (95% CI = 1.341–1.389). Simultaneously, one mm increase of weekly cumulative rainfall below 75 mm increased the risk of HFMD by 0.3% (CI = 1.002–1.003). While above 75 mm the effect was opposite and each mm increases of rainfall decreased the incidence by 0.5% (CI = 0.995–0.996). We also found that a difference between minimum and maximum temperature greater than 7°C elevated the risk of HFMD by 41% (CI = 1.388–1.439).

**Conclusion:**

Our findings suggest a strong association between HFMD and weather. However, the exact reason for the association is yet to be studied. Information on maximum temperature above 32°C and moderate rainfall precede HFMD incidence could help to control and curb the up-surging trend of HFMD.

## Introduction

Hand foot and mouth disease (HFMD) is caused by a number of different enteroviruses, among which are *enterovirus 71* (*EV71*) and *coxsackie A16* (*CA16*). The disease is transmitted through direct contact with respiratory droplets, feces, and blister fluid of infective patients or through contact with contaminated environment such as water, food, or surface [1], [2]. HFMD inflicts mainly children with mild clinical symptoms include fever, blisters and sores in mouth, palms and soles following 3–7 days of incubation period and a patient generally recovers in 7–10 days. Nevertheless, severe health consequences or death may occur owing to complications such as encephalitis, aseptic meningitis, and acute flaccid paralysis mainly following *EV71* infection. HFMD can be asymptomatic and it is possible for a recovered person to be infected again by different serotypes of enteroviruses [1]. There is no specific treatment or vaccine available; therefore, preventive measures such as avoid direct contact with infective patients, disinfection of viral contaminated items or premises, and good personal hygiene practices remain the only effective methods to disrupt disease transmission.

In recent decade, Asian countries have experienced increasing trend of HFMD outbreak with deaths among children due to severe complications [3], [4], [5]. HFMD has raised public health concerns in Asia following severe outbreaks in Malaysia and Taiwan in 1997 and 1998, respectively. About 6000 young children were infected with 42 deaths during the HFMD outbreak in Malaysia [6]. Whereas a total of 129,106 cases were reported in Taiwan with 405 children suffered complications that led to 78 deaths [7], [8]. In the first half of 2010, mainland China experienced 72% increase in HFMD cases compared with 2009; resulting in approximately 1.3 million reported cases [5]. Also, 5454 severe cases that caused 260 deaths were reported in mainland China between January to the first week of May [5]. HFMD outbreaks generally occur in 2–3 years cyclical pattern in endemic countries across the Western Pacific Region and *EV71*-related HFMD outbreak frequency is expected to increase in the region partly owing to complex factors including continued evolution and emergence of novel recombinants of *EV71*, inadequate healthcare capacity, and a lack of effective surveillance system in some countries [9].

HFMD is endemic in Singapore with *EV71* and *CA16* as two main dominant circulating strains. About 90% of the reported cases were children below 10 years old. The number of HFMD outbreaks reported from childcare centers, kindergartens, and preschools had escalated from about 167 in year 2001 to more than 1700 in year 2007. In the same period, the incidence rate among children aged 0–4 years surged from approximately 16 to 60 per 1000 populations and from 3 to 21 per 1000 populations among children 5–9 years old [10].

HFMD is endemic in the tropical and subtropical countries with tendency of higher number of cases in wet season depending on geographical locations [5], [11]. Whereas outbreaks usually occur in summer or early fall in temperate countries. Enterovirus surveillance in USA for the period 1970–2005 showed that *EV71* and *CA16* had endemic circulation pattern and that around 70% of cases were reported during warmer seasons between June–October [12]. Considering the seasonal pattern of HFMD outbreaks, we hypothesized that short-term changes in weather can influence the transmission dynamic of HFMD. This study aims to establish a relationship, analyze the effects and estimate potential thresholds of weekly temperature and rainfall associated with the risk of HFMD outbreaks in Singapore.

## Methods

Singapore is an island state nation with land size of approximately 710 km^2^ and population density of 7000 persons per km^2^ [13]. The island experiences tropical climate with high temperature, humidity, and rainfall.

Weekly cases of HFMD for the period 2001–2008 were obtained from the Weekly Infectious Diseases Bulletins of Communicable Diseases Division, Ministry of Health Singapore [14]. Report of HFMD cases from physicians, education institutions, and laboratories was mandatory in Singapore since October 2000 [15], [16]. Data of daily temperature and rainfall were retrieved from National Climatic Data Center, National Oceanic and Atmospheric Administration (NOAA), USA [17]. Weekly average temperature and cumulative rainfall were computed or aggregated from daily weather data. Weekly temperature difference (Tp) was computed as the difference between weekly average maximum and minimum temperature.

We established time series Poisson regression models to analyze the relationship between weather and HFMD cases adjusting for long-term time trends and seasonality. Time trends, driven by other factors such as circulating virus serotypes, disease control measures, and social behavior, could confound the relationship between temperature (e.g. seasonality) and HFMD. Therefore, trend and seasonality were adjusted to account for time varying factors that were influential on weekly HFMD incidence during the study period. We modeled the seasonality and long-term time trends in one function allowing the seasonality (driven by unknown factors) of HFMD to change between years as that allows more flexible adjustment for the potential confounding. We used smooth functions of natural cubic splines allowing 12 degrees of freedom (*df*) to adjust for seasonality and long-term time trends. Sensitivity to the effect estimates the flexibility of the smooth function of time trends was further tested using *df* of 8, 10, 15, and 20. The sensitivity tests did not show significantly different results of the estimated risk functions. We tested the lag time between temperature, rainfall, and HFMD by including lag terms 1–2 and 3–4 weeks using a backward stepwise model fitting procedure. The tests indicated insignificant results for lag term 3–4 weeks; thus, only lag term 1–2 weeks was included in the model. We analyzed the risk of HFMD as function of temperature and rainfall using 2 different models. In Model A, we included temperature difference (Tp) as the only temperature parameter; whereas both weekly minimum and maximum temperature were included in Model B. Model A was included to demonstrate risk function without potential co-linearity bias due to correlation between minimum and maximum temperature. To test the sensitivity of Model B for the co-linearity between minimum and maximum temperature, we repeated analysis using either minimum or maximum temperature parameter in each test. At a first stage, we allowed a non-linear exposure-response relationship using natural cubic splines with 4 *df*. In a second stage, we estimated the risk ratio of the relationship between weather parameters and HFMD incidence using piecewise linear spline functions where such approximation appeared applicable. One characteristic of infectious disease is the serial correlation between past and current incidence. Examination of the time series using autocorrelation function (ACF) indicated serial correlation for consecutive lags of HFMD cases; thus, we included autoregressive term of time lag 1–2 weeks based on average infectious and recovery period of a patient [1], [18].

Model A =  model with temperature differences and rainfall
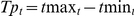


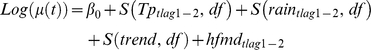



Model B  =  model with minimum temperature, maximum temperature, and rainfall
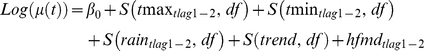



Where *Log (µ(t))* is the mean predicted weekly cases of HFMD; t equals the week in year 2001–2008; *β_0_* represents the intercept; *tmax_t_* represents maximum temperature; *tmin_t_* represents minimum temperature; *Tp_t_* means temperature difference; S denotes a cubic spline function with corresponding degrees of freedom (*df*); *rain_t_* is the cumulative rainfall; trend equals to week number running from 1 in the first week of year 2001; and *hfmd_t_* is an autoregressive term of HFMD.

The optimal model and parameters were selected and validated using post estimation residuals and the Akaike's information criterion (AIC). The post estimation was performed by plotting predicted residuals against observed data in addition to the partial autocorrelation function (PACF), scatter plots, normality tests and histogram of residuals. The risk ratio of HFMD incidence was presented as function of weather in relation to a minimum point of the curve or as corresponding to a unit increase of a particular weather predictor. All estimates were presented with corresponding 95% confidence intervals. Statistical analyses were conducted using R 2.10.1 [19] and STATA 11.1 (StataCorp, USA).

## Results

During the study period, Singapore experienced nationwide HFMD outbreaks in March-May in year 2002 and 2005–2008. Bimodal outbreaks occurred yearly with second outbreak in August-October during 2005 to 2008.

**Figure 1:**
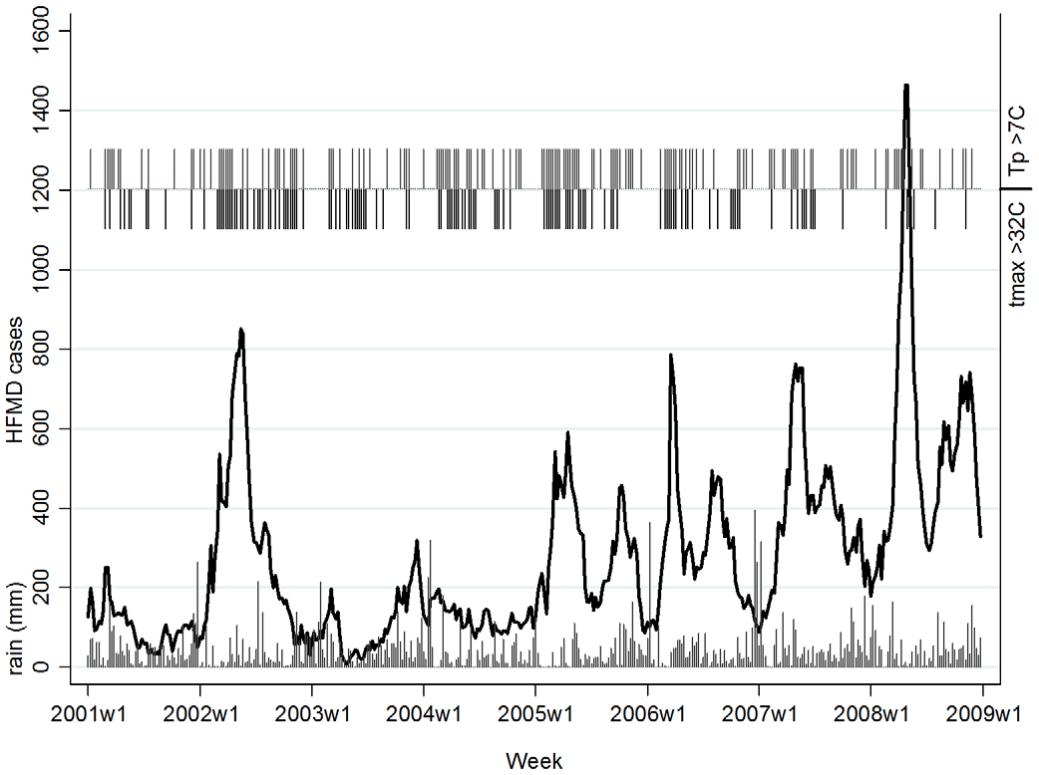
Time series of weekly HFMD cases, cumulative rainfall (rain), maximum temperature (tmax) >32°C, and temperature difference (Tp)>7°C in year 2001–2008.

Minimum and maximum temperature during the study period ranged from 22.8–27.6°C and 27.7–34.6°C, respectively. The highest weekly maximum temperature and lowest minimum temperature was recorded in 2005 and 2008 respectively. Overall, Singapore experienced temperature above 32°C in one-third of the study period with a total of 30 weeks in 2002 and about 6 weeks in 2008 (Figure 1). A total of 30 weeks with temperature difference (Tp) above 7°C was observed in year 2005, and highest weekly Tp of 8.8°C was recorded in 2006 and 2008. The weekly cumulative rainfall during the study period ranged from 0–388 mm with higher amount of rainfall recorded between October and January. About 80% of the weekly cumulative rainfall was below 75 mm.

**Figure 2:**
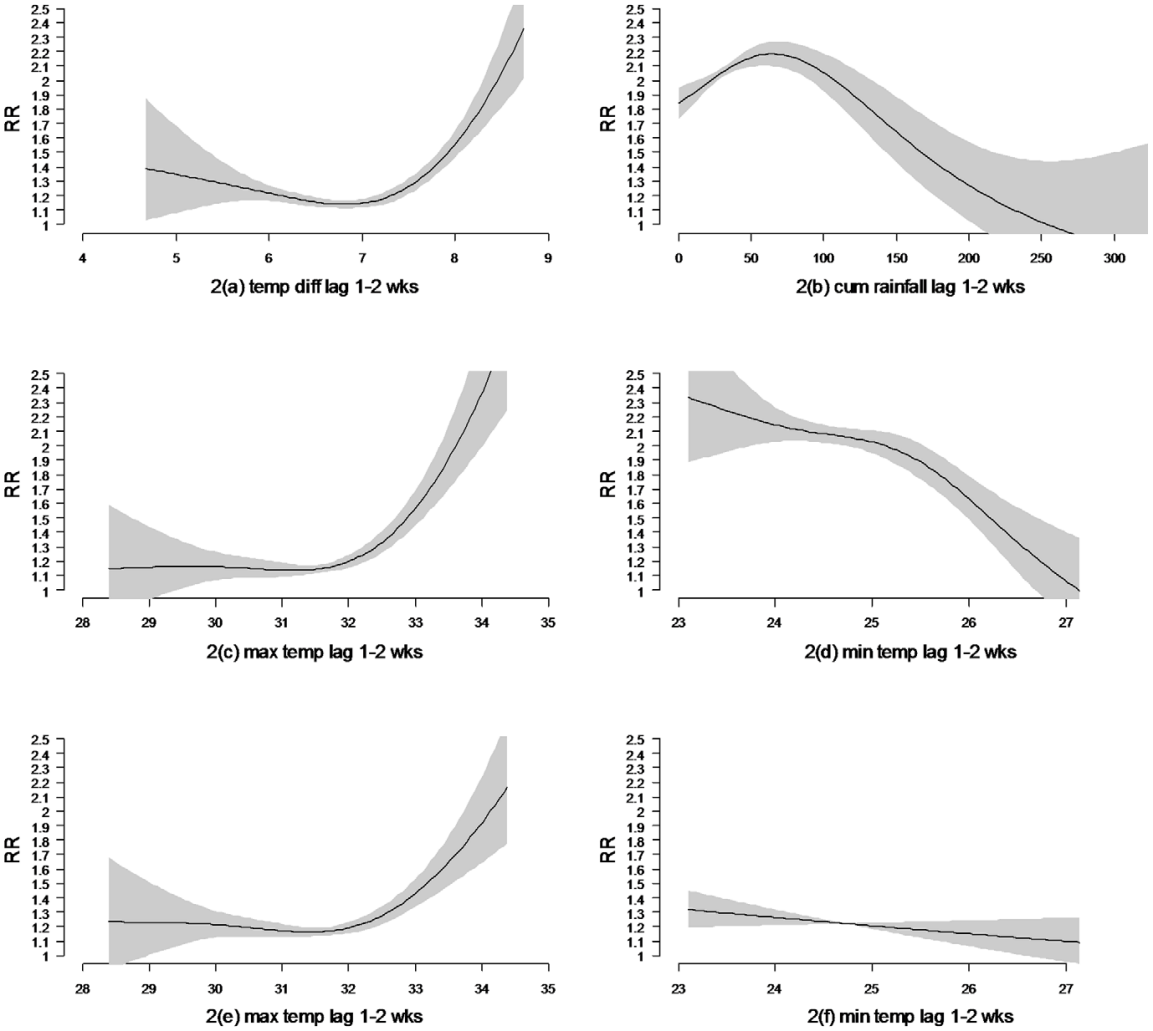
a–f: Relationship between HFMD incidence and weather. Relative risks of HFMD incidence as function of weekly (2a) temperature difference (Tp) & (2b) weekly cumulative rainfall at time lag of 1–2 weeks using model A; (2c) maximum temperature & (2d) minimum temperature at time lag of 1–2 weeks using model B; (2e) maximum temperature tested singly & (2f) minimum temperature tested singly at time lag of 1–2 weeks using model B (Shaded area: confidence interval).

The HFMD incidence was significantly associated with short term variability of weekly temperature difference (Tp), minimum temperature, maximum temperature, and cumulative rainfall at time lag of 1–2 weeks. Figure 2a depicts a wide J-shaped relationship between HFMD incidence and Tp with significant increase in the risk of HFMD incidence when Tp is above 7°C. Likewise, the risk of HFMD incidence rises steeply when maximum temperature is above 32°C; while low maximum temperature poses negligible effects on HFMD (Figure 2c). Inverse relationship is observed between minimum temperature and HFMD incidence (Figure 2d). Similar results are obtained when we test minimum and maximum temperature independently, except that minimum temperature is not statistically significant (Figure 2e & 2f). Figure 2b indicates the relative risk of HFMD incidence increases linearly with weekly cumulative rainfall between 0–75 mm; whereas every unit increases beyond 75 mm reduces the risk of HFMD incidence. During the study period, Tp>7°C mainly coincided with maximum temperature above 32°C and low rainfall, except year 2008.

In the piecewise linear Poisson regression function, we used breakpoints of 32°C for maximum temperature, 25°C for minimum temperature, 7°C for Tp, and 75 mm for rainfall. Table 1 shows every 1°C increases in maximum temperature above 32°C and in weekly Tp above 7°C elevates the risks of HFMD incidence significantly by 36% and 41%, respectively. The risk, however, declines by 17% with each degree increases in minimum temperature above 25°C. Additionally, a unit (mm) increases in weekly cumulative rainfall between 0–75 mm elevates risk of HFMD incidence by 0.3%.

**Table 1:** Risk of HFMD incidence as functions of weekly temperature difference (Tp), maximum temperature, minimum temperature, and rainfall.

Risk of HFMD incidence for an increase of one unit of	IRR	P<|z|	95% CI
Maximum temperature if the			
max temp < = 32°C	1.004	0.536	0.991 1.017
max temp >32°C	1.365	0.000	1.341 1.389
Minimum temperature if the			
min temp <25°C	0.972	0.017	0.949 0.995
min temp > = 25°C	0.833	0.000	0.816 0.851
Difference between minimum & maximum temperature if the			
temp difference < = 7°C	0.899	0.000	0.883 0.914
temp difference >7°C	1.414	0.000	1.388 1.439
Cumulative rainfall if the			
rain<75 mm	1.003	0.000	1.002 1.003
rain> = 75 mm	0.995	0.000	0.995 0.996

Observed and predicted time series of HFMD using both models produced similar results. Both model A and B explained about 87% of variations among HFMD cases using temperature, rainfall, and trend parameters. The residuals plots indicated model residuals did not violate the statistical modeling assumptions. Furthermore, the PACF showed no indication of further non-adjusted residual autocorrelation.

## Discussion

To date, very few studies document the link between weather and HFMD and none (that the authors are aware of) have previously shown that there exists an explicit relationship with threshold effects between short-term changes in weather and the incidence of HFMD. Our findings showed that high weekly maximum temperature and a large difference of minimum and maximum temperature increased the risk of HFMD incidence in the subsequent 1–2 weeks. The risk functions of minimum and maximum temperature provide more insights into the J-shaped curve of the relation between temperature difference and HFMD cases. Moreover, moderate weekly cumulative rainfall partly sustained the HFMD endemic during the study period. A study on the effects of climate on Herpangina and HFMD in Japan indicates that higher temperature could have influenced the increase of Herpangina & HFMD incidence [20].

Exact reasons for the relationship between weather and HFMD are not known. During the study period, high temperature in Singapore generally accompanied with rainfall below 75 mm and thus provided warm and damp environment which was conducive for enteroviruses viability and HFMD transmission. Rainfall levels below 75 mm were more prevalent during the annual outbreak transmission season of HFMD, while rainfall above this threshold dominated in the wet season. This could potentially explain the contrasting risk ratios. On the other hand, lower ambient temperature and heavy downpour could help to interrupt the disease transmission partly through serving as a barrier for social gathering activity or contact with other children in public. Together with multiple risk factors including continued evolution and introduction of novel strains of *EV71*, the anticipated increasing extreme weather events or extreme temperature as a consequence of climate change may amplify the risks of HFMD outbreaks in the future.

Enteroviruses can tolerate temperature fluctuation and survive well in sewage treatment and water environment [2]. Thermal effect on enteroviruses vary depending on temperature sensitive or resistant strains of serotypes as well as the types, pH, evaporation rate, and water content of viral environment [21], [22], [23], [24]. A laboratory study on recovery of enteroviruses in various soil conditions shows that thermal effect plays an important role on the infectivity or plague forming units of enteroviruses; the study indicates enteroviruses in sand amended with septic tank liquor can be recovered in up to 36 and 11 days at 22°C and 37°C, respectively [22]. Other studies have also revealed that temperature resistant strains of *EV71* that link with fatal complications are not inactivated at 40°C [24], [25].

The insignificant result of minimum temperature when tested alone indicated that minimum temperature was not sufficient by itself in predicting risk of HFMD. Though maximum temperature performed well in predicting HFMD incidence, inclusion of minimum temperature could strengthen the predictive power of the model. This was likely due to difference in maximum and minimum temperature was influential on HFMD incidence as shown in figure 2a. Also, temperature difference was likely more influential on HFMD incidence in year 2008 as maximum temperature was below 32°C almost throughout the year.

Our approach controls for non-climatic determinants of HFMD such as infectious disease control exercises, good hygiene practices, school holiday, and dominant strains of circulating enterovirus in the trends function. Since 1998, Singapore set up surveillance system and formed multi-sector HFMD Task Force to plan, monitor, and manage outbreaks in Singapore [15]. Public health measures that aimed to prevent HFMD outbreaks included thorough disinfection of virus contaminated premises and temporary closure of educational institutions such as childcare centers/preschools/kindergartens where disease transmission took place for more than 15 days [26]. Different dominating serotypes of enterovirus had been alternating during outbreaks in the period 2001 to 2008. *CA16* was the dominant circulating virus during the outbreaks in year 2002, 2005, and 2007; whereas *EV71* was the predominating strain for the epidemics in 2006 and 2008 [10], [26]. It was reported that *EV71* affected significantly more cases during 2008 outbreaks, compared with 2006 [26].

HFMD cases were lower during school holiday in June, November, and December. Separation of children during the school holiday reduced social contacts; thus, interrupted transmission in the childcare centers and led to reducing HFMD incidence. During the outbreak of deadly episodes of severe acute respiratory syndrome (SARS) in year 2003, school and childcare centers were closed for 2 additional weeks in March in response to concerned parents. Stringent preventive measures included daily body temperature taking in all schools across the nation and children with high body temperature were sent home. Additionally, children who had contact with suspected SARS patients or as siblings of contacts were advised to stay at home for minimum 10 days. Massive public education was launched to caution community to observe strict hygiene practices, avoid crowded areas, and other precautionary measures to prevent and disrupt the disease transmission of SARS. Though the outbreak of SARS was recorded between the months from March to May, the ripple effects of the preventive measures and the alertness among populations could partly be the reasons for lower HFMD incidence in this year.

The bimodal outbreaks in years 2005–2008 coincided mostly with weekly maximum temperature greater than 32°C and temperature difference above 7°C. Nonetheless, the second peak which occurred between 2–6 months after the first outbreak could also possibly be influenced by the extension of disease transmission from the first outbreak due to continual presence of viruses in the environment [26]. Enterovirus can remain in an infected person's feces for several weeks after onset of symptoms and also possibly persist for days or weeks on materials found in domestic or institution environment [18], [23], [27], [28]. The high population density in Singapore could also compound the disease transmission rate and sustainability of outbreak. Furthermore, the infective dose of coxsackie viruses requires for human is low; therefore, transmission of HFMD persists even with the presence of low amount of shed virus [23].

Asymptomatic and unreported cases are common limitations encountered in the study of infectious diseases epidemiology. It could limit and bias the analysis as actual cases could be many times higher. The disease transmission pattern is also less clearly defined as asymptomatic patients represent an undetected source of infection. Furthermore, heavy traffic flow among populations and trade between Singapore and neighbor countries could also influence the size and duration of outbreaks in Singapore during study period.

In view of the links between warmer season and HFMD cases, climate change and global warming may increase susceptibility of areas or regions to the transmission of HFMD, which may possibly be another important emerging infectious disease affecting millions of life. It was reported that seasonal patterns of enteroviruses differed by geographical localities and that some tropical and subtropical countries experienced more outbreaks in the rainy season [29]. Thus, we encourage similar studies in diverse geographical areas to increase understanding of the impacts of weather on HFMD cases, as well as studies to establish the causal associations between meteorological determinants and pathways of HFMD infection.

### Conclusion

We found a strong relationship between HFMD incidence and the preceding 1–2 weeks weather parameters after adjusting for other time varying factors. A maximum daily temperature above 32°C and rainfall up to 75 mm is expected to increase the HFMD incidence in the subsequent 1–2 weeks. These findings suggest that weather parameters can be used as early risk indicators for potential HFMD outbreaks. Implementing a simple weather-based early warning could help 1) local authority to heighten alert, intensify surveillance, and activate infection control measures to prevent or curb disease outbreaks; and 2) community to exercise vigilance and take precautionary actions such as good hygiene practices and isolation to disrupt HFMD transmission chain. However, future studies are required to confirm this relationship in other regions. Subsequent studies need to elucidate the chain of events following temperature and rainfall changes and their pathways to the increase of HFMD incidence.
